# Effect of Li Adsorption on the Electronic and Hydrogen Storage Properties of Acenes: A Dispersion-Corrected TAO-DFT Study

**DOI:** 10.1038/srep33081

**Published:** 2016-09-09

**Authors:** Sonai Seenithurai, Jeng-Da Chai

**Affiliations:** 1 Department of Physics, National Taiwan University, Taipei 10617, Taiwan; 2 Center for Theoretical Sciences and Center for Quantum Science and Engineering, National Taiwan University, Taipei 10617, Taiwan

## Abstract

Due to the presence of strong static correlation effects and noncovalent interactions, accurate prediction of the electronic and hydrogen storage properties of Li-adsorbed acenes with *n* linearly fused benzene rings (*n* = 3–8) has been very challenging for conventional electronic structure methods. To meet the challenge, we study these properties using our recently developed thermally-assisted-occupation density functional theory (TAO-DFT) with dispersion corrections. In contrast to pure acenes, the binding energies of H_2_ molecules on Li-adsorbed acenes are in the ideal binding energy range (about 20 to 40 kJ/mol per H_2_). Besides, the H_2_ gravimetric storage capacities of Li-adsorbed acenes are in the range of 9.9 to 10.7 wt%, satisfying the United States Department of Energy (USDOE) ultimate target of 7.5 wt%. On the basis of our results, Li-adsorbed acenes can be high-capacity hydrogen storage materials for reversible hydrogen uptake and release at ambient conditions.

Hydrogen (H_2_) is a pure energy carrier with high energy content in terms of mass, and is highly abundant on the earth in the form of water. Therefore, hydrogen is considered as the next-generation clean and green fuel which could replace fossil fuels. However, the efficient production, storage, and transportation of hydrogen are to be achieved for hydrogen-based economy. Among them, the storage of hydrogen has recently posed a great challenge to the scientific community, due to the difficulty in achieving a safe and efficient storage system which could store large amounts of hydrogen in a container of small volume, light weight, and low cost^1^,^2^,^3^,^4^,^5^,^6^,^7^,^8^.

In 2015, the United States Department of Energy (USDOE) set the 2020 target of 5.5 wt% and the ultimate target of 7.5 wt% for the gravimetric storage capacities of onboard hydrogen storage materials for light-duty vehicles^8^. As of now, there have been several methods for storing hydrogen, such as physical storage methods where hydrogen is stored in vessels at very high pressures (e.g., 350 to 700 bar), cryogenic methods where hydrogen is stored at very low temperatures (e.g., 20 K), chemical storage in the form of metal hydrides, and adsorption-based storage in high surface area materials^1^,^2^,^3^,^4^,^5^,^6^,^7^. Experimentally, none of these methods has achieved the gravimetric and volumetric storage capacities set by the USDOE with fast kinetics, while some theoretical studies have reported materials with the ideal storage capacities. For reversible hydrogen adsorption and desorption at ambient conditions (298 K and 1 bar), the ideal binding energies of H_2_ molecules on hydrogen storage materials should be in the range of about 20 to 40 kJ/mol per H_2_^9^,^10^,^11^.

To achieve the USDOE target, high surface area materials, such as graphene, carbon nanotubes, and metal-organic frameworks (MOFs), have been extensively studied in recent years. However, as these materials bind H_2_ molecules weakly, they work properly only at low temperatures. For ambient storage applications, it is essential to increase the binding energies of H_2_ molecules on these materials to the aforementioned ideal range^9^,^10^,^11^, and hence various novel methods are being explored. Generally adopted methods are substitution doping (by B, N, etc.) and adatom adsorption (by Li, Al, Ca, Ti, etc.), just to name a few^3^,^12^,^13^,^14^,^15^,^16^. Among them, Li adsorption is particularly attractive, because of its light weight with which a high gravimetric storage capacity could be easily achieved. In addition, as Li may adsorb H_2_ molecules strongly through a charge-transfer induced polarization mechanism^2^,^17^,^18^,^19^, the resulting hydrogen binding energy could lie in the desirable range. Consequently, a number of works have been devoted to hydrogen storage in Li-adsorbed materials^13^,^15^,^20^,^21^,^22^,^23^,^24^,^25^,^26^,^27^,^28^,^29^,^30^,^31^,^32^,^33^.

Over the past two decades, organic semiconductors have received considerable attention from many researchers, owing to their potential role in molecular electronics, photonics, and photovoltaics. Among them, linear *n*-acenes (C_4*n*+2_H_2*n*+4_), consisting of *n* linearly fused benzene rings (see Fig. 1(a)), have recently attracted much attention due to their unique electronic properties^34^,^35^,^36^,^37^,^38^,^39^,^40^,^41^. Note that *n*-acenes can be attractive for hydrogen storage applications, due to their quasi-one-dimensional structures and the feasibility of synthesis of shorter acenes^42^,^43^. Recent interest in the development of organic field-effect transistors (OFETs) and photovoltaics has made rapid progress in acene-based crystals (e.g., tetracene, pentacene, and hexacene crystals)^44^. For example, highly oriented crystals of 6,13-bis(triisopropylsilylethynyl)pentacene (TIPS pentacene) have been experimentally realized at large scales for designing OFETs^45^. Pentacene thin films have also been synthesized, which could be hydrogen storage materials, if metal atoms can be properly intercalated as in graphite^46^. Also, because of the immense interest in the development of Li-ion battery, Li intercalation has been experimentally feasible for some carbon-based materials in recent years.

Even though there has been a keen interest in developing acene-based electronics and Li intercalation technique, the studies of electronic and hydrogen storage properties of Li-adsorbed *n*-acenes (see Fig. 1(b–e)) are quite limited. Experimentally, it was argued that there is a significant decrease in the stability of longer *n*-acenes (*n* > 5), hindering the synthesis of these materials^42^. However, the longer acenes and acene derivatives have been synthesized in some matrices^43^,^47^, which also serve as the building blocks of novel MOFs and other three-dimensional crystals^11^,^42^,^44^,^48^. Therefore, once efficient synthesis is available for the crystallization of *n*-acenes, Li could be subsequently intercalated to synthesize Li-adsorbed *n*-acenes. Theoretically, due to the multi-reference character of ground-state wavefunctions, the properties of longer *n*-acenes cannot be adequately described by conventional electronic structure methods, including the very popular Kohn-Sham density functional theory (KS-DFT)^49^ with conventional (i.e., semilocal^50^, hybrid^51^,^52^,^53^, and double-hybrid^54^,^55^,^56^,^57^) density functionals^58^. High-level *ab initio* multi-reference methods are typically needed to accurately predict the properties of longer *n*-acenes^34^,^36^,^59^. However, as the number of electrons in *n*-acene, 26*n* + 16, quickly increases with the increase of *n*, there have been very scarce studies on the properties of longer *n*-acenes using multi-reference methods, due to their prohibitively high cost.

Recently, we have developed thermally-assisted-occupation density functional theory (TAO-DFT)^35^,^38^, an efficient electronic structure method for the study of large ground-state systems (e.g., containing up to a few thousand electrons) with strong static correlation effects^39^,^40^,^41^. Interestingly, TAO-DFT has similar computational cost as KS-DFT, and reduces to KS-DFT in the absence of strong static correlation. Very recently, we have studied the electronic properties of zigzag graphene nanoribbons (ZGNRs) using TAO-DFT, where the strong static correlation effects have been properly described^39^. Accordingly, TAO-DFT can be an ideal electronic structure method for studying the electronic properties of Li-adsorbed *n*-acenes. Besides, the orbital occupation numbers in TAO-DFT can be useful for examining the possible multi-reference character of Li-adsorbed *n*-acenes^35^,^38^,^39^,^40^,^41^. In addition, for the hydrogen storage properties, as the interaction between H_2_ and Li-adsorbed *n*-acenes may involve dispersion (van der Waals) interactions, electrostatic interactions, and orbital interactions^3^,^10^,^60^, the inclusion of dispersion corrections^61^,^62^ in TAO-DFT can be essential to properly describe noncovalent interactions. Therefore, in this work, we adopt dispersion-corrected TAO-DFT^38^ to study the electronic and hydrogen storage properties of Li-adsorbed *n*-acenes with various chain lengths (*n* = 3–8).

## Computational Details

All calculations are performed with a development version of Q-Chem 4.3^63^. Results are computed using TAO-BLYP-D^38^ (i.e., TAO-DFT with the dispersion-corrected BLYP-D exchange-correlation density functional^61^ and the LDA *θ*-dependent density functional 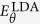 (see Eq. (41) of ref. 35)) at the fictitious temperature *θ* = 7 mhartree (as defined in ref. 35). For all the calculations, we adopt the 6–31G(d) basis set with the fine grid EML(75,302), consisting of 75 Euler-Maclaurin radial grid points and 302 Lebedev angular grid points. For the interaction energies of the weakly bound systems (e.g., Li binding energy, H_2_ binding energy, etc.), the counterpoise correction^64^ is employed to reduce the basis set superposition error (BSSE).

## Results and Discussion

### Electronic Properties

To start with, we obtain the ground state of *n*-acene (*n* = 3–8), by performing spin-unrestricted TAO-BLYP-D calculations for the lowest singlet and triplet energies of *n*-acene on the respective geometries that were fully optimized at the same level of theory. The singlet-triplet energy (ST) gap of *n*-acene is calculated as (*E*_T_ − *E*_S_), the energy difference between the lowest triplet (T) and singlet (S) states. Similar to previous findings^34^,^35^,^36^,^38^,^39^, the ground states of *n*-acenes are found to be singlets for all the chain lengths investigated (see Fig. 2).

**Figure 1:**
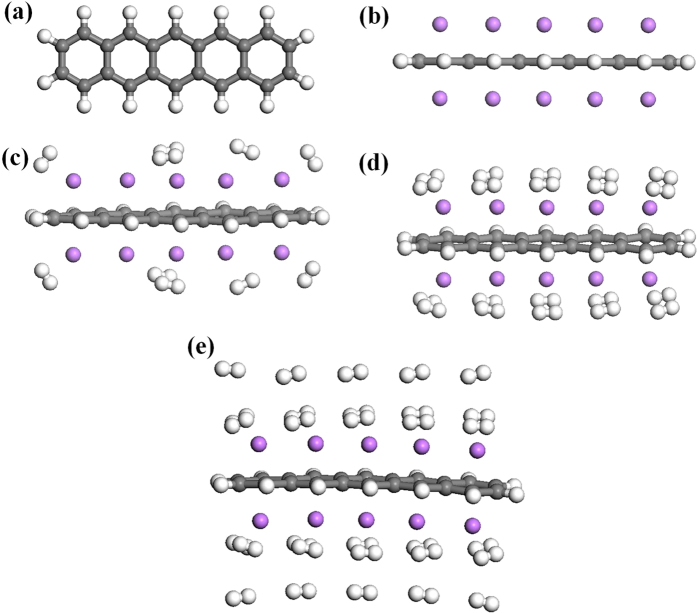
Structures of (**a**) pure 5-acene, (**b**) Li-adsorbed 5-acene, (**c**) Li-adsorbed 5-acene with one H_2_ molecule adsorbed on each Li adatom, (**d**) Li-adsorbed 5-acene with two H_2_ molecules adsorbed on each Li adatom, and (**e**) Li-adsorbed 5-acene with three H_2_ molecules adsorbed on each Li adatom. Here, grey, white, and purple balls represent C, H, and Li atoms, respectively.

Next, at the ground-state (i.e., the lowest singlet state) geometry of *n*-acene, we place *n* Li atoms on one side of *n*-acene, and *n* Li atoms on the other side (i.e., at high coverage). To obtain the most stable adsorption site, Li atoms are initially placed on different possible sites, such as the hexagon site (the center of a benzene ring), the top site (the top of a C atom), the bridge site (the midpoint of C-C bond), and the edge site (the edge of *n*-acene), and the structures are subsequently optimized. As illustrated in Fig. 1(b), the hexagon site is the most stable adsorption site. The isolated form of Li atoms is found to be preferred over clustering^33^, which may be attributed to the quasi-one-dimensional nature of *n*-acene. Therefore, in this work, Li-adsorbed *n*-acene is regarded as *n*-acene-2*n*Li, which is *n*-acene with 2*n* Li atoms adsorbed on all the hexagon sites.

**Figure 2:**
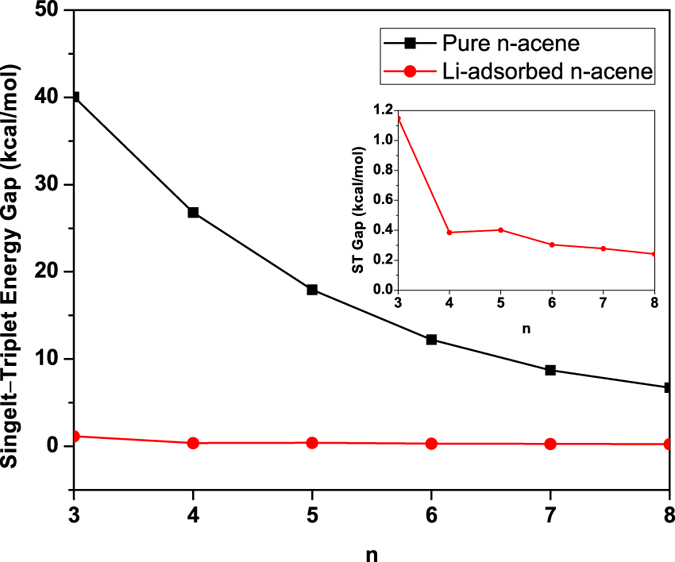
Singlet-triplet energy (ST) gap of pure/Li-adsorbed *n*-acene as a function of the chain length, calculated using TAO-BLYP-D. The inset shows a close-up view for the ST gap of Li-adsorbed *n*-acene.

Similar to the procedure described above, the ST gap of Li-adsorbed *n*-acene is also computed. As shown in Fig. 2, the ST gap of Li-adsorbed *n*-acene generally decreases with increasing chain length. The ground states of Li-adsorbed *n*-acenes remain singlets for all the chain lengths studied. Owing to the presence of Li adatoms, the ST gap of Li-adsorbed *n*-acene is much smaller than that of pure *n*-acene.

Due to the symmetry constraint, the spin-restricted and spin-unrestricted energies for the lowest singlet state of pure/Li-adsorbed *n*-acene calculated using the exact theory, should be identical^35^,^37^,^38^,^39^,^40^,^41^. To assess the possible symmetry-breaking effects, spin-restricted TAO-BLYP-D calculations are also performed for the lowest singlet energies on the respective optimized geometries. Within the numerical accuracy of our calculations, the spin-restricted and spin-unrestricted TAO-BLYP-D energies for the lowest singlet state of pure/Li-adsorbed *n*-acene are essentially the same (i.e., essentially no unphysical symmetry-breaking effects occur in our spin-unrestricted TAO-BLYP-D calculations).

**Figure 3:**
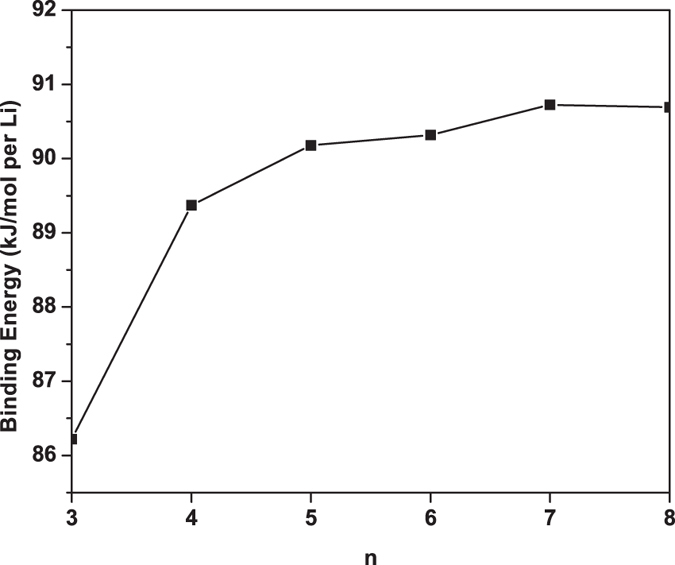
Li binding energy on *n*-acene as a function of the chain length, calculated using TAO-BLYP-D.

To examine the energetic stability of adsorbed Li atoms, the Li binding energy, *E*_*b*_(Li), on *n*-acene is calculated by


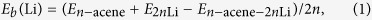


where *E*_*n*-acene_ is the total energy of *n*-acene, *E*_2*n*Li_ is the total energy of the 2*n*Li adatoms on the hexagon sites, and *E*_*n*-acene-2*n*Li_ is the total energy of Li-adsorbed *n*-acene. *E*_*b*_(Li) is subsequently corrected for BSSE using a standard counterpoise correction, where the *n*-acene is considered as one fragment, and the 2*n*Li adatoms are considered as the other fragment. As shown in Fig. 3, *n*-acene can strongly bind the Li adatoms with the binding energy range of 86 to 91 kJ/mol per Li.

**Figure 4:**
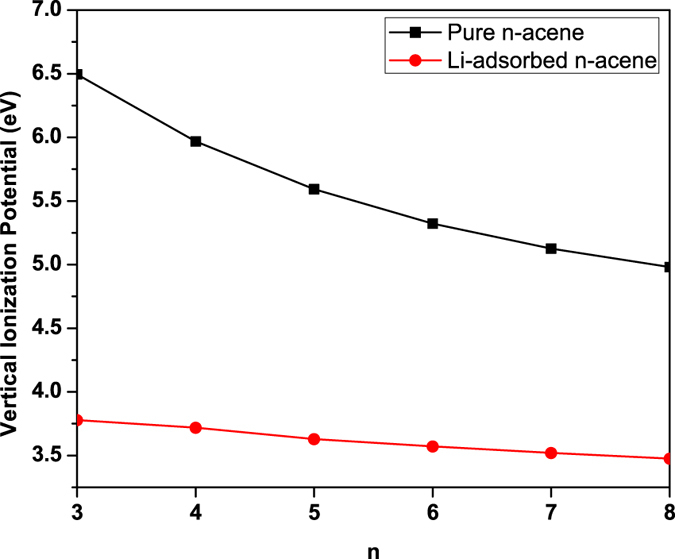
Vertical ionization potential for the lowest singlet state of pure/Li-adsorbed *n*-acene as a function of the chain length, calculated using TAO-BLYP-D.

**Figure 5:**
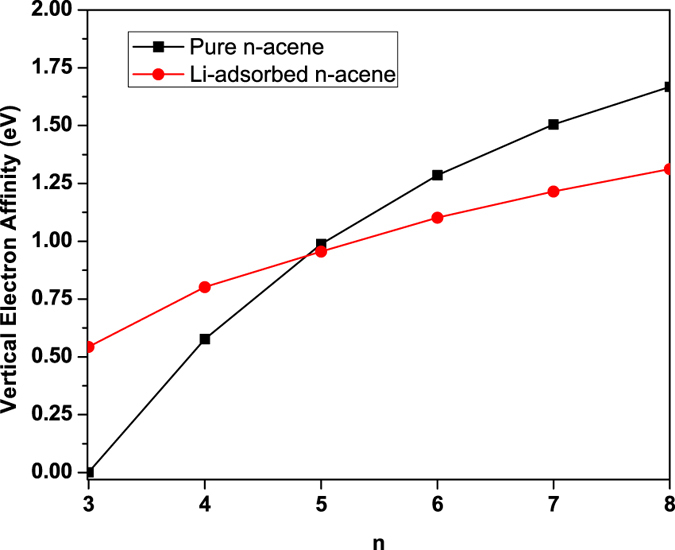
Vertical electron affinity for the lowest singlet state of pure/Li-adsorbed *n*-acene as a function of the chain length, calculated using TAO-BLYP-D.

**Figure 6:**
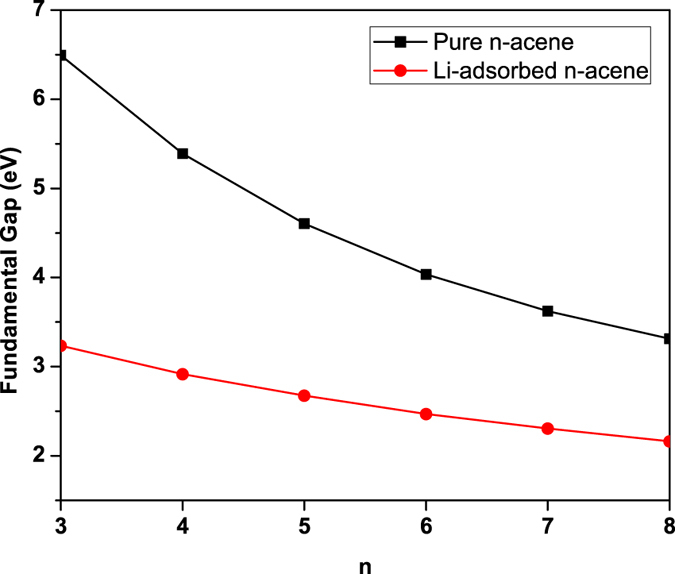
Fundamental gap for the lowest singlet state of pure/Li-adsorbed *n*-acene as a function of the chain length, calculated using TAO-BLYP-D.

At the ground-state (i.e., the lowest singlet state) geometry of pure/Li-adsorbed *n*-acene, containing *N* electrons, the vertical ionization potential IP_*v*_ = *E*_*N*−1_ − *E*_*N*_, vertical electron affinity EA_*v*_ = *E*_*N*_ − *E*_*N*+1_, and fundamental gap *E*_*g*_ = IP_*v*_ − EA_*v*_ = *E*_*N*+1_ + *E*_*N*−1_ − 2*E*_*N*_ are obtained with multiple energy-difference calculations, where *E*_*N*_ is the total energy of the *N*-electron system. With increasing chain length, IP_*v*_ monotonically decreases (see Fig. 4), EA_*v*_ monotonically increases (see Fig. 5), and hence *E*_*g*_ monotonically decreases (see Fig. 6). As shown, the IP_*v*_, EA_*v*_, and *E*_*g*_ values of Li-adsorbed *n*-acene are less sensitive to the chain length than those of pure *n*-acene. Note also that the *E*_*g*_ value of Li-adsorbed *n*-acene (*n* = 4–8) is within the most interesting range (1 to 3 eV), giving promise for applications of Li-adsorbed *n*-acenes in nanophotonics.

**Figure 7:**
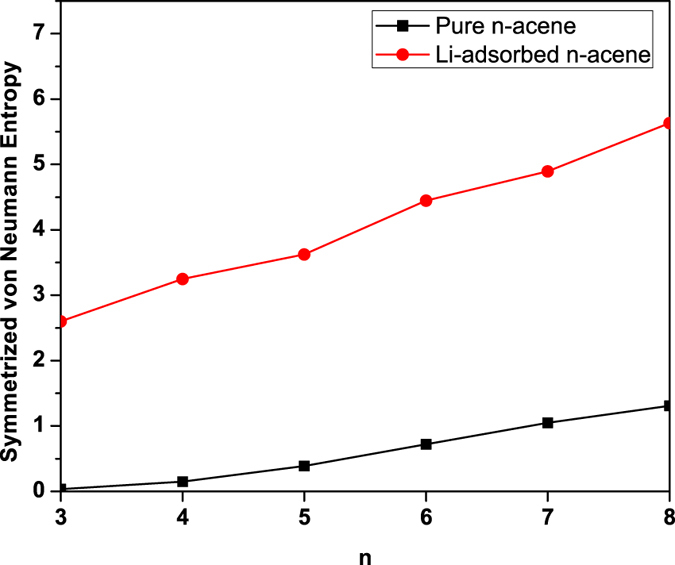
Symmetrized von Neumann entropy for the lowest singlet state of pure/Li-adsorbed *n*-acene as a function of the chain length, calculated using TAO-BLYP-D.

To assess the possible multi-reference character of pure/Li-adsorbed *n*-acene, we calculate the symmetrized von Neumann entropy^37^,^38^,^39^,^41^


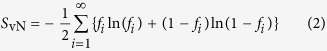


for the lowest singlet state of pure/Li-adsorbed *n*-acene as a function of the chain length. Here, *f*_*i*_ the occupation number of the *i*^th^ orbital obtained with TAO-BLYP-D, ranging from 0 to 1, is approximately equal to the occupation number of the *i*^th^ natural orbital^35^,^38^. Note that *S*_vN_ provides insignificant contributions for a single-reference system ({*f*_*i*_} are close to either 0 or 1), and rapidly increases with the number of active orbitals ({*f*_*i*_} are fractional for active orbitals, and are close to either 0 or 1 for others). As shown in Fig. 7, *S*_vN_ monotonically increases with the chain length. Therefore, the multi-reference character of pure/Li-adsorbed *n*-acene increases with the chain length.

On the basis of several measures (e.g., the smaller ST gap, the smaller *E*_*g*_, and the larger *S*_vN_), Li-adsorbed *n*-acene should possess stronger multi-reference character than pure *n*-acene for each *n*. Consequently, KS-DFT with conventional density functionals should be insufficient for the accurate description of the properties of Li-adsorbed *n*-acene. Besides, as accurate multi-reference calculations are prohibitively expensive for the longer pure/Li-adsorbed *n*-acene, the use of TAO-DFT in this study is well justified.

### Hydrogen Storage Properties

As pure carbon-based materials bind H_2_ molecules weakly (i.e., mainly governed by dispersion interactions), they are not ideal hydrogen storage materials at ambient conditions^9^. Similarly, pure *n*-acenes are not promising for ambient storage applications, as the binding energies of H_2_ molecules remain small. Besides, the number of H_2_ molecules that can be adsorbed on each benzene ring is limited, due to the repulsive interaction between the adsorbed H_2_ molecules at short distances^65^. Therefore, the more the adsorbed H_2_ molecules, the less the average H_2_ binding energy on *n*-acene. Consequently, pure *n*-acenes cannot be high-capacity hydrogen storage materials at ambient conditions.

Here, we examine the hydrogen storage properties of Li-adsorbed *n*-acene (*n* = 3–8). As illustrated in Fig. 1, at the ground-state geometry of Li-adsorbed *n*-acene, *x* H_2_ molecules (*x* = 1–3) are initially placed on different possible sites around each Li adatom, and the structures are subsequently optimized to obtain the most stable geometry. All the H_2_ molecules are found to be adsorbed molecularly. The average H_2_ binding energy, *E*_*b*_(H_2_), on Li-adsorbed *n*-acene is calculated by


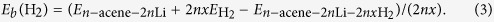


**Figure 8:**
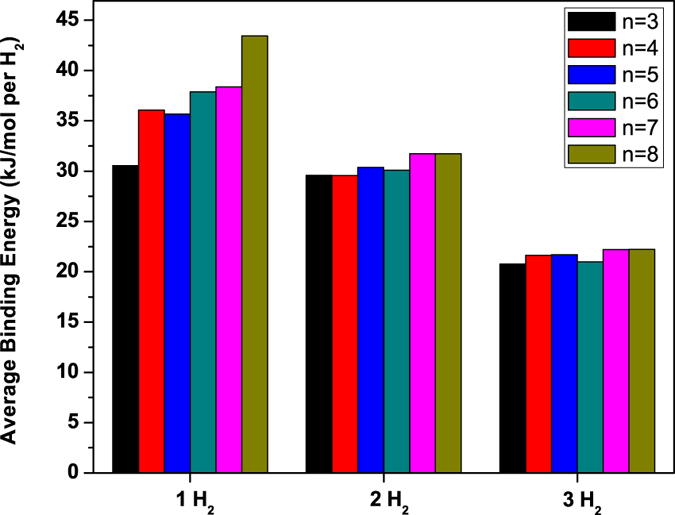
Average H_2_ binding energy on Li-adsorbed *n*-acene (*n* = 3–8) as a function of the number of H_2_ molecules adsorbed on each Li adatom, calculated using TAO-BLYP-D.

Here, 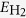 is the total energy of a free H_2_ molecule, and 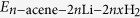 is the total energy of Li-adsorbed *n*-acene with *x* H_2_ molecules adsorbed on each Li adatom. *E*_*b*_(H_2_) is subsequently corrected for BSSE using a standard counterpoise correction. As shown in Fig. 8, *E*_*b*_(H_2_) is in the range of 31 to 43 kJ/mol per H_2_ for *x* = 1, in the range of 30 to 32 kJ/mol per H_2_ for *x* = 2, and in the range of 21 to 22 kJ/mol per H_2_ for *x* = 3, falling in the ideal binding energy range.

Here, we examine if the binding energies of successive H_2_ molecules are in the ideal binding energy range (i.e., not just the average H_2_ binding energy). The binding energy of the *y*^th^ H_2_ molecule (*y* = 1–3), *E*_*b*,*y*_(H_2_), on Li-adsorbed *n*-acene is calculated by


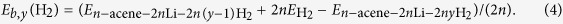


**Figure 9:**
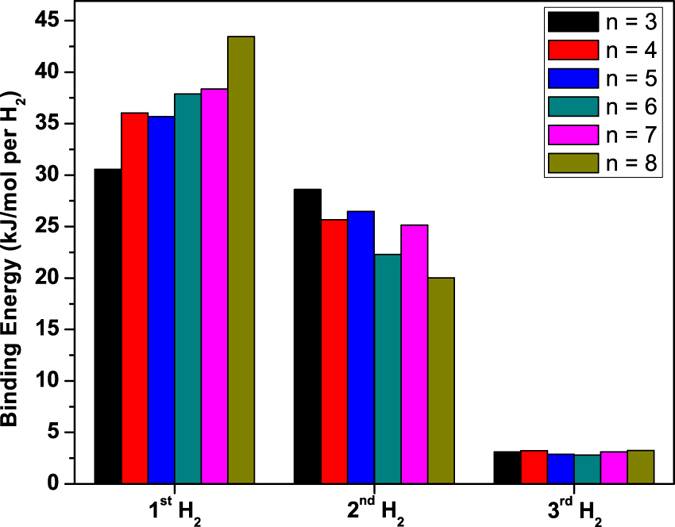
Binding energy of the *y*^th^ H_2_ molecule (*y* = 1–3) on Li-adsorbed *n*-acene (*n* = 3–8), calculated using TAO-BLYP-D.

Similarly, *E*_*b*,*y*_(H_2_) is subsequently corrected for BSSE using a standard counterpoise correction. As shown in Fig. 9, *E*_*b*,1_(H_2_) is in the range of 31 to 43 kJ/mol per H_2_, *E*_*b*,2_(H_2_) is in the range of 20 to 29 kJ/mol per H_2_, and *E*_*b*,3_(H_2_) is about 3 kJ/mol per H_2_. Therefore, while the first and second H_2_ molecules can be adsorbed on Li-adsorbed *n*-acene in the ideal binding energy range, the third H_2_ molecule is only weakly adsorbed (i.e., not suitable for ambient temperature storage).

For practical applications, we estimate the desorption temperature, *T*_*D*_, of the adsorbed H_2_ molecules using the van’t Hoff equation^12^,^15^,


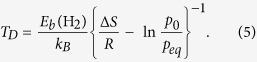


Here, *E*_*b*_(H_2_) is the average H_2_ binding energy (see Eq. (3)), Δ*S* is the change in hydrogen entropy from gas to liquid phase (Δ*S* = 13.819*R* taken from ref. 66), *p*_0_ is the standard atmospheric pressure (1 bar), *p*_*eq*_ is the equilibrium pressure, *k*_*B*_ is the Boltzmann constant, and *R* is the gas constant. As shown in Table 1, *T*_*D*_ for Li-adsorbed *n*-acene (*n* = 3–8) with *x* H_2_ molecules (*x* = 1–2) adsorbed on each Li adatom, is estimated using Eq. (5) at *p*_*eq*_ = 1.5 bar (as adopted in ref. 9) and at *p*_*eq*_ = 1 bar (the standard atmospheric pressure). For Li-adsorbed *n*-acene (except for *n* = 3), the *T*_*D*_ values are slightly higher than room temperature for *x* = 1, and slightly lower than room temperature for *x* = 2. Even for Li-adsorbed 3-acene, the *T*_*D*_ values remain close to room temperature. Therefore, Li-adsorbed *n*-acenes can be viable hydrogen storage materials at ambient conditions.

As Li-adsorbed *n*-acene (*n* = 3–8) can bind up to 4*n* H_2_ molecules (i.e., each Li adatom can bind up to two H_2_ molecules) with the average and successive H_2_ binding energies in the ideal range, the corresponding H_2_ gravimetric storage capacity, *C*_*g*_, is evaluated by


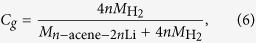


where *M*_*n*-acene-2*n*Li_ is the mass of Li-adsorbed *n*-acene, and 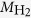 is the mass of H_2_. As shown in Table 1, *C*_*g*_ is in the range of 9.9 to 10.7 wt%, satisfying the USDOE ultimate target of 7.5 wt%. Based on the observed trends, at the polymer limit (*n* → ∞), the *C*_*g*_ value of Li-adsorbed polyacene can be estimated as 11.2 wt%, very close to that of Li-adsorbed *n*-acene (*n* = 3–8). However, the USDOE target value refers to the complete storage system, including the storage material, enclosing tank, insulation, piping, etc.^8^, rather than the storage material alone. Therefore, the *C*_*g*_ values obtained here may not be directly compared to the USDOE target. The real *C*_*g*_ value will depend on the design of the complete storage system, and hence the comparison has to be made after considering all of these issues. Nonetheless, as the *C*_*g*_ values obtained here are much higher than the USDOE ultimate target, the complete storage systems based on Li-adsorbed *n*-acenes are likely to be high-capacity hydrogen storage materials at ambient conditions.

## Conclusions

In conclusion, we have studied the electronic properties (i.e., the Li binding energies, ST gaps, vertical ionization potentials, vertical electron affinities, fundamental gaps, and symmetrized von Neumann entropy) and hydrogen storage properties (i.e., the average and successive H_2_ binding energies, H_2_ desorption temperatures, and H_2_ gravimetric storage capacities) of Li-adsorbed *n*-acenes (*n* = 3–8) using our recently developed TAO-DFT with dispersion corrections. Since Li-adsorbed *n*-acenes have been shown to exhibit stronger multi-reference character than pure *n*-acenes, KS-DFT with conventional density functionals can be unreliable for studying the properties of these systems. Besides, accurate multi-reference calculations are prohibitively expensive for the longer Li-adsorbed *n*-acenes, and hence the use of TAO-DFT in this study is well justified. On the basis of our results, Li-adsorbed *n*-acenes can bind up to 4*n* H_2_ molecules (i.e., each Li adatom can bind up to two H_2_ molecules) with the average and successive H_2_ binding energies in the ideal range of about 20 to 40 kJ/mol per H_2_. Consequently, for Li-adsorbed *n*-acenes, the H_2_ desorption temperatures are close to room temperature, and the H_2_ gravimetric storage capacities are in the range of 9.9 to 10.7 wt%, satisfying the USDOE ultimate target of 7.5 wt%. Therefore, Li-adsorbed *n*-acenes could serve as high-capacity hydrogen storage materials for reversible hydrogen uptake and release at ambient conditions.

On the basis of our results, it is possible to place Li on both sides of the acene molecule. However, for acene crystals (i.e., real materials), it can be challenging to place Li on both side of the acene molecule, as the acene molecules are stacked against each other. To resolve this, we may follow the proposal of Deng *et al.*^21^, and consider Li-adsorbed pillared acenes, where the intermolecular distance of acene molecules can be properly increased to provide sufficiently large space for Li and H_2_. A systematic study of the electronic and hydrogen storage properties of Li-adsorbed pillared acenes is essential, and may be considered for future work. In addition, it should be noted that Li-doped systems could have the following practical issues: the preoccupancy of Li sites by solvent molecules, low stability against air and water, etc., which are open to experimentalists^33^.

We hope that our results will guide experimental studies for developing and synthesizing reversible hydrogen storage materials. Owing to recent advances in dispersion-corrected TAO-DFT, the search for ideal hydrogen storage materials can be readily extended to large systems with strong static correlation effects (i.e., systems beyond the reach of conventional electronic structure methods). In the future, we intend to address how the electronic and hydrogen storage properties vary with different adatoms (e.g., Al, Ca, Ti, etc.) and underlying carbon-based materials (e.g., graphene nanoribbons, nanoflakes, etc.).

## Additional Information

**How to cite this article**: Seenithurai, S. and Chai, J.-D. Effect of Li Adsorption on the Electronic and Hydrogen Storage Properties of Acenes: A Dispersion-Corrected TAO-DFT Study. *Sci. Rep.* **6**, 33081; doi: 10.1038/srep33081 (2016).

## Figures and Tables

**Table 1:** Average H_2_ binding energy *E*_*b*_(H_2_) (kJ/mol per H_2_), H_2_ desorption temperature *T*_*D*_ (K), and H_2_ gravimetric storage capacity *C*_*g*_ (wt%) for Li-adsorbed *n*-acene (*n* = 3–8) with *x* H_2_ molecules (*x* = 1–2) adsorbed on each Li adatom, calculated using TAO-BLYP-D.

*n*	*E*_*b*_(H_2_)		*T*_*D*_ (*p*_*eq*_ = 1.5)		*T*_*D*_ (*p*_*eq*_ = 1)		*C*_*g*_
	1 H_2_	2 H_2_	1 H_2_	2 H_2_	1 H_2_	2 H_2_	
3	30.55	29.58	258	250	266	258	9.9
4	36.06	30.87	305	261	314	269	10.2
5	35.69	31.08	302	263	311	270	10.4
6	37.87	30.08	320	254	330	262	10.5
7	38.36	31.75	324	269	334	276	10.6
8	43.45	31.73	368	268	378	276	10.7

Here, *T*_*D*_ is estimated using the van’t Hoff equation (see Eq. (5)) at *p*_*eq*_ = 1.5 (bar) and at *p*_*eq*_ = 1 (bar), and *C*_*g*_ (see Eq. (6)) is calculated only for *x* = 2.
